# Glycyrrhizic Acid and Its Derivatives: Promising Candidates for the Management of Type 2 Diabetes Mellitus and Its Complications

**DOI:** 10.3390/ijms231910988

**Published:** 2022-09-20

**Authors:** Dechao Tan, Hisa Hui Ling Tseng, Zhangfeng Zhong, Shengpeng Wang, Chi Teng Vong, Yitao Wang

**Affiliations:** Macau Centre for Research and Development in Chinese Medicine, State Key Laboratory of Quality Research in Chinese Medicine, Institute of Chinese Medical Sciences, University of Macau, Macau 999078, China

**Keywords:** glycyrrhizic acid, glycyrrhetinic acid, ammonium glycyrrhizinate, diammonium glycyrrhizinate, type 2 diabetes mellitus, diabetic complications, insulin resistance, glucose tolerance

## Abstract

Type 2 diabetes mellitus (T2DM) is a chronic metabolic disease, which is characterized by hyperglycemia, chronic insulin resistance, progressive decline in β-cell function, and defect in insulin secretion. It has become one of the leading causes of death worldwide. At present, there is no cure for T2DM, but it can be treated, and blood glucose levels can be controlled. It has been reported that diabetic patients may suffer from the adverse effects of conventional medicine. Therefore, alternative therapy, such as traditional Chinese medicine (TCM), can be used to manage and treat diabetes. In this review, glycyrrhizic acid (GL) and its derivatives are suggested to be promising candidates for the treatment of T2DM and its complications. It is the principal bioactive constituent in licorice, one type of TCM. This review comprehensively summarized the therapeutic effects and related mechanisms of GL and its derivatives in managing blood glucose levels and treating T2DM and its complications. In addition, it also discusses existing clinical trials and highlights the research gap in clinical research. In summary, this review can provide a further understanding of GL and its derivatives in T2DM as well as its complications and recent progress in the development of potential drugs targeting T2DM.

## 1. Introduction

Diabetes mellitus (DM) is a chronic metabolic disease which is characterized by a persistent excess of glucose in the blood, known as hyperglycemia, and it leads to severe and irreparable damage in different organs over time, especially the heart, blood vessels, eyes, kidneys, and nerves [1,2]. International Diabetes Federation pointed out that about 537 million adults (20–79 years old) are living with DM in 2021, of which 3 in 4 live in low-and middle-income countries, and more than 95% of DM patients are type 2 DM (T2DM) patients. Moreover, the total number of people living with DM is expected to increase to 643 million by 2030 and 783 million by 2045 [1,3]. T2DM is caused by a combination of two primary factors: defective insulin secretion by pancreatic β-cells and chronic insulin resistance in insulin-sensitive tissues [4]. Multiple drugs with hypoglycemic actions for the treatment of T2DM are available in the market nowadays, including sulfonylureas, meglitinides, biguanides, thiazolidinediones, α-glucosidase inhibitors, dipeptidyl peptidase-4 inhibitors, sodium-glucose transporter inhibitors, and glucagon-like peptide-1 (GLP-1) receptor agonists [5,6]. However, most of them have adverse effects, such as nausea, bloating, diarrhea, weight gain, stomach pain, hypoglycemia, and secondary failure of therapy, which may influence medication adherence in patients [5]. In addition, iminosugars, such as miglitol, were shown to be another promising therapeutic agent for T2DM [7,8], and studies were focused on the research and development of miglitol derivatives [9,10,11]. Moreover, T2DM patients are susceptible to develop into poorly controlled diabetic patients, who are highly associated with diabetic complications. As a result, there is no doubt that current conventional anti-diabetic drugs have certain limitations to the minimization of side effects and long-term glucose management, thus anti-T2DM drugs still need to move toward more comprehensive and accessible treatments.

Licorice, the dried root and rhizome of *Glycyrrhiza uralensis*, *Glycyrrhiza inflata* or *Glycyrrhiza glabra* in the Fabaceae family, is a traditional herbal medicine which is extensively used in Europe and Asia [12,13]. Glycyrrhizic acid (GL), also known as glycyrrhizin, is the principal bioactive constituent in licorice and exists as two isomers, 18α-GL and 18β-GL, which make up 5–9% of licorice. Moreover, the aqueous extracts of licorice contain 10–20% GL [14,15]. Studies have shown that GL has many pharmacological effects, including anti-inflammatory [16,17], anti-oxidative [18,19], anti-viral [20,21], immunoregulatory [22,23], anti-cancer [24,25], and anti-diabetic [26,27] activities. In particular, GL exerts therapeutic effects on T2DM, such as reducing blood glucose levels [28], decreasing serum insulin levels [29], enhancing insulin sensitivity [30,31], improving glucose tolerance and homeostasis [28,32,33], and regulating lipid metabolism [33,34,35]. Meanwhile, GL also displays beneficial effects on diabetic complications, including cardiovascular disease [36], cerebrovascular disease [37,38], diabetic nephropathy [39,40], diabetic neuropathy [41], and diabetic retinopathy [31,42,43].

To the best of our knowledge, there is no review summarizing the therapeutic effects and related mechanisms of GL and its derivatives in T2DM. Therefore, we conducted a comprehensive review of GL and its derivatives for the treatment of T2DM and its complications. The related literatures were retrieved from PubMed, Web of Science, and Google Scholar. The search algorithm for PubMed was as follows: (“glycyrrhizic acid” OR “glycyrrhetinic acid” OR “ammonium glycyrrhizinate” OR “diammonium glycyrrhizinate” OR “dipotassium glycyrrhizinate”) AND (“diabetes” OR “insulin” OR “lipid”) with no other restrictions. Similar but adapted search terms were also used for other search engines. Through a comprehensive reading of the retrieved literatures, we have excluded literatures of Type 1 DM, review articles, and other non-related articles. After exclusion, 54 articles were selected, which were related to the treatment of T2DM by GL and its derivatives. Firstly, we provide an overview of GL and its derivatives. Then, we focus on how GL and its derivatives exert therapeutic effects on T2DM and its complications. Finally, we discuss the clinical applications of GL in T2DM patients and provide clinical perspectives. Therefore, this comprehensive review can provide a further understanding of GL and its derivatives in T2DM as well as its complications and recent progress in the development of potential drugs targeting T2DM.

## 2. GL and Its Derivatives

**Figure 1:**
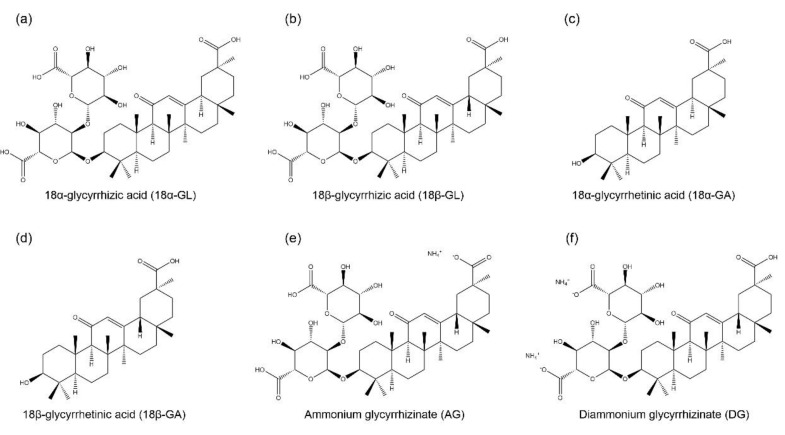
Chemical structures of glycyrrhizic acid (GL) and its derivatives. They include two isomers of glycyrrhizic acid, namely, (**a**) 18α-glycyrrhizic acid (18α-GL) and (**b**) 18β-glycyrrhizic acid (18β-GL); two isomers of glycyrrhetinic acid (GA), namely, (**c**) 18α-glycyrrhetinic acid (18α-GA) and (**d**) 18β-glycyrrhetinic acid (18β-GA); (**e**) ammonium glycyrrhizinate (AG); and (**f**) diammonium glycyrrhizinate (DG).

GL is an amphiphilic compound, its hydrophilic part is represented by the glucuronic acid residues, and its hydrophobic part is the glycyrrhetinic acid (GA) residue [44]. GA is not only a hydrolytic product of GL, but it is also one of the main bioactive components of licorice with two isoforms, 18α-GA and 18β-GA [45,46]. GL and its aglycon, GA, have promising therapeutic effects, which have attracted the researchers to develop them into clinical medicine, including anti-cancer, anti-inflammatory, anti-viral, anti-diabetic, and hepatoprotective effects [47,48]. However, studies found that the oral administration of GL is poorly absorbed from the gut, but it is slowly converted into 18β-GA under intestinal bacterial hydrolysis, and the hydrolysis product 18β-GA is absorbed in the gut [49,50]. Therefore, due to the poor bioavailability of GL, it has been designed to conjugate with various metals or ammonium to form water-soluble salts for improving its bioavailability, including ammonium glycyrrhizinate (AG), diammonium glycyrrhizinate (DG), and dipotassium glycyrrhizinate [51,52,53]. The structures of GL, GA, AG, and DG are shown in Figure 1.

## 3. Anti-Diabetic Mechanisms of GL and Its Derivatives in T2DM

**Figure 2:**
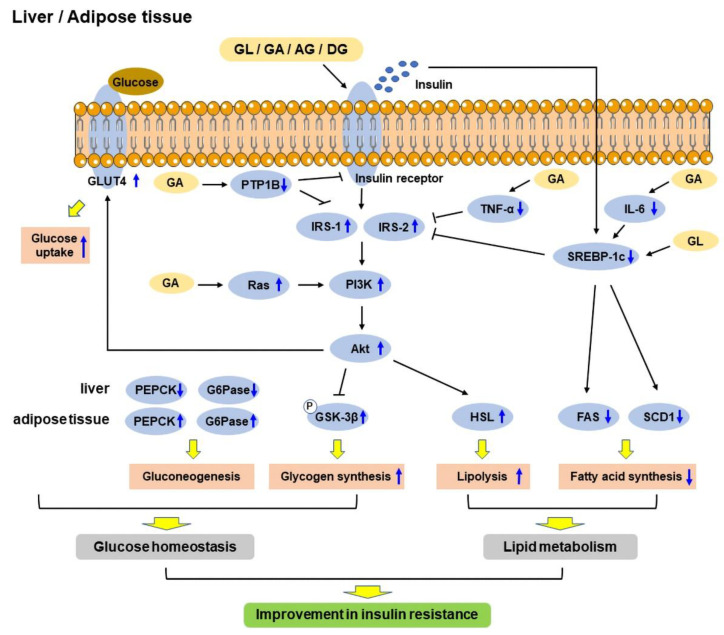
Schematic diagram showing the mechanisms of glycyrrhizic acid (GL) and its derivatives in improving insulin resistance in the liver and adipose tissue. GL and its derivatives act on the insulin receptor to regulate gluconeogenesis and enhance glycogen synthesis via PI3K/Akt signaling pathway, and increase glucose uptake via GLUT4, thus regulating glucose homeostasis. On the other hand, they increase lipolysis through PI3K/Akt/HSL pathway and reduce fatty acid synthesis through downregulating SREBP-1c/FAS/SCD1 pathway, thereby regulating lipid metabolism. Therefore, GL and its derivatives improve insulin resistance through improving glucose homeostasis and lipid metabolism. FAS: Fatty acid synthetase; G6Pase: Glucose-6-phosphatase; GLUT4: Glucose transporter 4; GSK-3β: Glycogen synthase kinase-3β; HSL: Hormone-sensitive lipase; IGF-1: Insulin-like growth factor 1; IL-6: Interleukin 6; IRS-1: Insulin receptor substrate 1; IRS-2: Insulin receptor substrate 2; PEPCK: Phosphoenolpyruvate carboxykinase; PI3K: Phosphoinositide 3-kinase; PTP1B: Protein tyrosine phosphatase 1B; SCD1: Stearoyl CoA desaturase 1; SREBP-1c: Sterol regulatory element-binding protein 1c; TNF-α: Tumor necrosis factor-α.

T2DM is mainly characterized by hyperglycemia, which eventually results in chronic insulin resistance in insulin-sensitive tissues and defective insulin secretion by pancreatic β-cells [4]. The anti-diabetic effects of GL and its derivatives have been confirmed in vitro and in vivo. The mechanisms include improving glucose tolerance [33] and insulin sensitivity [28], regulating glucose homeostasis [28] and lipid metabolism [54], and enhancing insulin secretion [55]. A schematic diagram shows the mechanisms of GL and its derivatives in improving insulin resistance in the liver and adipose tissue (Figure 2).

### 3.1. Insulin Resistance

Insulin is a hormone produced by the pancreas that controls glucose levels in the blood and helps in storing glucose in the insulin-sensitive organs, including skeletal muscle, fat, and the liver, where it is used to provide energy. The inability of responding to insulin in insulin-sensitive metabolic tissues, known as insulin resistance, is one of the main pathophysiology of T2DM, thus the tissues cannot uptake glucose from the blood easily. As a result, the pancreas produces more insulin to help in reducing blood glucose levels [56,57]. At a later stage, the pancreatic β-cells cannot compensate for the high demand of insulin; therefore, this eventually leads to β-cell dysfunction and defect in insulin secretion, and hyperglycemia occurs [58]. Studies have shown that GL, GA, and DG could improve insulin sensitivity in diabetic rodents [28,30,31,33,54,59,60,61,62,63,64,65,66,67,68].

There are several indicators of insulin resistance, including homeostasis model assessment of insulin resistance (HOMA-IR), homeostatic model assessment of β-cells (HOMA-β), and quantitative insulin sensitivity check index (QUICKI) [69]. HOMA was developed by Matthews et al., and it is used to quantify insulin resistance and β-cell function from fasting blood glucose and insulin levels [70], while QUICKI is another similar mathematical transformation from fasting blood glucose and insulin levels [71]. A study found that GL reduced HOMA-IR index [28,54,60,63,64,65,66], and increased HOMA-β and QUICKI indices [63] in diabetic rodents, indicating that GL could improve insulin sensitivity. Moreover, impaired fasting blood glucose levels is another indicator of insulin resistance and is characterized by a notable increase in hepatic insulin resistance [72]. GL and 18β-GA were demonstrated to reduce fasting blood glucose levels in diabetic rodents [28,33,54,59,60,63,65,66,67]. In addition, high fasting serum insulin levels is another sign of insulin resistance [73], and GL was found to decrease fasting serum insulin levels in diabetic rodents [28,33,54,66,67].

The insulin sensitivity of metabolic tissues plays a vital role in reducing blood glucose levels, and these tissues mainly include liver, adipose tissue, and skeletal muscle [74]. White adipose tissue (WAT) is an endocrine organ, which stores energy as triacylglycerols, and secretes hormones to regulate glucose homeostasis and adipokines to regulate inflammation. It was suggested that hypertrophy in WAT is associated with insulin resistance [75]. High-fat diet (HFD) was shown to increase visceral adipocyte size and induce adipose tissue inflammation in HFD-induced obese rats. In contrast, GL could decrease the size of adipocytes, thereby reducing body and WAT weights [30]. Moreover, it also reduced the adipocyte size and area in the subcutaneous WAT of rats [65,68]. Similarly, GL and DG could reduce the weights of WAT in HFD-induced obese rodents [30,61]. Furthermore, peroxisome proliferator-activated receptor-γ (PPAR-γ) is a nuclear receptor, which is highly expressed in adipose tissue [76]. The activation of PPAR-γ has been shown to upregulate the expressions of genes that are related to insulin signaling, thus enhancing insulin sensitivity [77]. Studies found that the GL treatment improved insulin sensitivity via upregulating the expression of PPAR-γ in the WAT and skeletal muscle [28,54,59]. On the other hand, the liver is responsible for glucose uptake and lipid synthesis and metabolism [78]. HFD increased liver weight, and GL reduced the liver weight and hepatic steatosis in HFD-induced diabetic mice [33].

Insulin receptor (IR) is a transmembrane receptor, which can be activated by insulin and insulin-like growth factor (IGF). IR and insulin receptor substrate (IRS) play a vital role in regulating glucose homeostasis in the pancreas and insulin-sensitive tissues [56]. IRS acts as a secondary messenger to transmit signals from insulin to downstream intracellular pathways, thus enhancing insulin sensitivity and the transcription of insulin-related genes [79]. GL was found to increase IR mRNA expression and its phosphorylation to enhance insulin sensitivity [30,31]. In addition, another study found that GL enhanced insulin sensitivity through the phosphorylation of IRS-1 and IRS-2 in HFD-induced diabetic mice [33]. Moreover, protein tyrosine phosphatase 1B (PTP1B), the main enzyme involved in IR desensitization, regulates insulin and leptin levels [80]. It is a negative regulator of insulin signaling pathway, and its inhibitors have become an attractive strategy to treat T2DM and obesity [81]. Interestingly, 18α-GA and 18β-GA were identified as competitive PTP1B inhibitors [82,83]. Two other derivatives of GA, namely, indole- and N-phenylpyrazole-GA, also showed potent non-competitive inhibition of PTP1B [84].

Insulin-like growth factor-1 (IGF-1) is a growth hormone that can enhance glucose uptake and reduce hepatic glucose production, thereby improving insulin sensitivity [85]. Insulin and IGF-1 regulate many signaling pathways, including Ras/mitogen-activated protein kinase (MAPK), and phosphoinositide 3-kinase (PI3K)/Akt pathway [56]. The activation of Ras is involved in the development of T2DM, and the inactivation of Ras genes improved insulin sensitivity [86]. In addition, PI3K/Akt pathway was identified as one of the vital pathways that are associated with insulin resistance [87]. GA was shown to improve glucose uptake and reverse insulin resistance by targeting Ras proteins and activating PI3K/Akt pathway, respectively [62]. Moreover, studies found that advanced glycation end products (AGE)-receptor for AGE (RAGE) axis activated oxidative stress and pro-inflammatory pathways, which contributed to insulin resistance [88,89]. GL was found to improve insulin resistance and downregulate RAGE expression spontaneously, suggesting that GL might improve insulin sensitivity via suppressing AGE-RAGE axis [60].

GLP-1, a 30-amino acid peptide hormone, is secreted by gut enteroendocrine cells, which improve insulin resistance in adipocytes [90]. It is currently used as a T2DM therapeutic. In addition, the inhibition of intestinal farnesoid X-activated receptor (FXR) or FXR deficiency was shown to promote GLP-1 secretion [91], and DG was also found to improve insulin resistance and glucose tolerance through downregulation of FXR [61].

Studies have shown that inflammation is associated with insulin resistance, and the most vital pro-inflammatory mediators include tumor necrosis factor-α (TNF-α) and interleukin 6 (IL-6). The inhibition of pro-inflammatory mediators was suggested to be one of the strategies for preventing the development of insulin resistance and the pathogenesis of diabetes [92,93,94,95,96]. GA was shown to reduce fasting plasma TNF-α and IL-6 levels to enhance insulin-responsive pathways [62].

### 3.2. Glucose Tolerance and Homeostasis

Glucose tolerance is another indicator of diabetes and defined as the ability of the body to dispose glucose. Impaired glucose tolerance is often occurred in T2DM, and the progression from normal to impaired glucose tolerance is caused by insulin resistance [97]. Studies have shown that GL could improve glucose tolerance in diabetic rodents [28,33,67].

Glucose homeostasis can be affected by many factors, including insulin, glucagon, concentration of free fatty acids (FFA), and nutritional factors [98]. Gluconeogenesis is the process of glucose production, and gluconeogenic enzymes play a vital role in this process, including phosphoenolpyruvate carboxykinase (PEPCK), fructose-1,6-bisphosphatase (FBPase), and glucose-6-phosphatase (G6Pase) [99]. Glycogenesis is the process of glycogen synthesis, in which glucose is converted and stored as glycogen in the liver, which is activated by insulin in response to high blood glucose levels. Pyruvate dehydrogenase (PDase), an essential enzyme in glycogen synthesis, is also a substrate of glycogen synthase kinase 3β (GSK-3β), and GSK-3β induces pyruvate dehydrogenase E1α (PDH-E1α) phosphorylation in response to insulin to promote glycogen synthesis [100]. 11β-hydroxysteroid dehydrogenase (11β-HSD) is an enzyme involved in the pathogenesis of metabolic syndrome, which is characterized by hyperglycemia, dyslipidemia, diabetes, and arterial hypertension, and has two isoforms, 11β-HSD1 and 11β-HSD2 [101]. 11β-HSD1 is highly expressed in insulin-sensitive tissues, including liver, WAT, and skeletal muscle [102]. The inhibition of 11β-HSD1 ameliorated hyperglycemia and improved insulin sensitivity in diabetic mice [32]. In addition, PEPCK is a critical enzyme in gluconeogenesis, and the improved activity of PEPCK leads to elevated glucose output and aggravation of diabetes, whereas the defects of PEPCK result in lethal hypoglycemia [103]. Hexose-6-phosphate dehydrogenase (H6PDH), an enzyme that is localized in the endoplasmic reticulum lumen, converts glucose-6-phosphate (G6P) and nicotinamide adenine dinucleotide phosphate (NADP) to produce nicotinamide adenine dinucleotide phosphate hydrogen (NADPH). It provides a high [NADPH]/[NADP^+^] ratio for 11β-HSD1 amplification of intracellular active glucocorticoid production [104]. Moreover, increased 11β-HSD1-dependent active glucocorticoid production is associated with insulin resistance, T2DM, and its cardiovascular complications [105].

Studies found that GL could reduce PEPCK and G6Pase mRNA expressions to suppress gluconeogenesis, and upregulate PDase and GSK-3β mRNA expressions to increase glycogen synthesis in the liver [30,33,66]. Similarly, GL reduced PEPCK activities in the liver and kidney of normal rats [65], and alleviated the over-activities of PEPCK and G6Pase in the liver and kidney in rats with metabolic syndrome [54]. In addition, GL could reduce H6PDH activities in the liver, kidney, WAT, and muscle [65,66]. Moreover, GL could decrease the activities of 11β-HSD1 and 11β-HSD2 in the liver, kidney, WAT, and muscle to reduce blood glucose levels [64,65,66]. Furthermore, two isoforms of GA have different inhibition activities on 11β-HSD; 18α-GA selectively inhibits 11β-HSD1, but not 11β-HSD2, while 18β-GA favorably inhibits 11β-HSD2 [106].

Glucose transporter type 4 (GLUT4) is an insulin-regulated glucose transporter, which is involved in rapid glucose uptake in various kinds of cells to regulate glucose homeostasis, and its downregulation is closely associated with the development of glucose tolerance [107,108,109]. GA was found to promote GLUT4 expression by targeting Ras protein to regulate MAPK pathway [62]. Similarly, GL elevated GLUT4 expression in the skeletal muscle of HFD-induced diabetic rats to improve glucose homeostasis [28]. Moreover, GL and GA were shown to enhance insulin-stimulated glucose uptake in 3T3L-1 adipocytes [55].

Due to the poor bioavailability of GL, it has been formulated as nanoparticles. GL-loaded nanoparticles improved lipid profile and lowered fasting blood glucose levels in nicotinamide plus streptozotocin (STZ)-induced T2DM rats. Interestingly, the dosages used in GL-loaded nanoparticles were only a quarter of the dosages of the pure GL form [110,111]. Moreover, a combination of GL-loaded nanoparticles and thymoquinone-loaded nanocapsules was applied, displaying better anti-diabetic activities than when administered separately in nicotinamide and STZ-induced T2DM rats, including decreased blood glucose levels and improved lipid profile [111]. Glycosylated hemoglobin A1c is another biochemical parameter that is used to estimate the severity of diabetes, and its high level is an indicator of poor control of blood glucose levels [112]. Moreover, GL-loaded nanoparticles decreased the level of glycosylated hemoglobin A1c [110,111]. Furthermore, GA, as an efficient liver-specific ligand, was made to be a liver-targeted drug delivery carrier and conjugated with chitosan lactate and poly(ethylene glycol) to form nanoparticles containing siRNA-CREB regulated transcription coactivator 2 (CRTC2). These nanoparticles were shown to be accumulated in the liver after 2 h of treatment, reduce blood glucose levels, and inhibit hepatic gluconeogenesis in rats [113,114].

### 3.3. Lipid Metabolism

The liver is responsible for the regulation of lipid metabolism, uptake of FFA, and export of fat [115]. In T2DM, lipid metabolism is altered, and fat uptake and delivery are imbalanced, which is linked with the over-production of very low-density lipoprotein (VLDL) [115,116]. Many studies found that HFD or high-fat-high-sucrose diet increased serum and hepatic triglyceride (TG), total cholesterol, low-density lipoprotein (LDL) cholesterol, and FFA levels, and decreased high-density lipoprotein (HDL) cholesterol levels in rodents [30,33,54,117,118]. In contrast, GL could improve serum lipid parameters, including decreases in serum and hepatic FFA, TG, total cholesterol, LDL cholesterol, and VLDL levels, but an increase in HDL cholesterol levels in diabetic rodents [30,33,54,68,110,111,117,118]. Similarly, GL-loaded nanoparticles could also decrease total cholesterol, TG, LDL, and VLDL levels, and increase HDL levels in diabetic rats [110]. Meanwhile, GL could also increase lipid uptake in cardiac and skeletal muscle [54].

Lipoprotein lipase (LPL) is a multi-functional glycoprotein enzyme, which plays a vital role in lipid parameters, such as the levels of TG, LDL cholesterol, HDL cholesterol, and VLDL, and its activity is upregulated by apolipoprotein C-II and downregulated by apolipoprotein C-III [119,120]. Increased LPL activity can be used as an indicator of enhanced lipid uptake into tissues [121]. GL was shown to upregulate LPL expression in the kidney, heart, muscle, and WAT [54,59,68,117]. In addition, other studies showed that GL promoted TG metabolism by inducing LPL activity, which upregulated hepatic apolipoprotein C-II expression and suppressed apolipoprotein C-III expression in HFD-induced diabetic mice [33].

Excess FFA and lipid droplets lead to intramuscular accumulation of lipid intermediates, which results in the inhibition of insulin signal transduction to impair insulin sensitivity [122]. GL was shown to decrease lipid deposition in the liver, kidney, heart, and muscle in HFD-induced diabetic rodents [33,117]. Interestingly, a study showed that GL treatment improved insulin sensitivity in HFD-induced obese rats, which might be through decreased lipid deposition [117]. Moreover, hepatic lipogenesis is regulated by a transcription factor, sterol regulatory element-binding transcription factor 1c (SREBP-1c), which is activated by insulin and regulates the genes that are involved in fatty acid and TG synthesis, including fatty acid synthetase (FAS) and stearoyl CoA desaturase (SCD) 1 [123]. GL was shown to improve lipid metabolism mainly through reducing hepatic lipogenesis and improving fatty acid metabolism, via downregulation of SREBP-1c, FAS, and SCD1 expressions, and upregulation of peroxisome proliferator-activated receptor-α (PPAR-α), carnitine palmitoyl transferase 1α (CPT1α), and acyl-coenzyme A dehydrogenase (ACADS), respectively [33]. GL and GA were also found to reduce the early stage of adipogenesis and lipid accumulation in 3T3-L1 adipocytes through downregulating CCAAT/enhancer-binding protein (C/EBP)-β and C/EBP-δ, PPAR-γ, and SCD expressions [55,124]. Similarly, 18β-GA was found to inhibit lipid accumulation, suppress adipogenic differentiation via inhibiting Akt phosphorylation and downregulating PPAR-γ and C/EBP-α expressions, and stimulate lipolysis via upregulation of adipose TG lipase (ATGL), hormone-sensitive lipase (HSL), and perilipin (Plin-1) expressions in 3T3-L1 adipocytes [35]. However, Yamamoto et al. showed that GL did not stimulate lipolysis in 3T3-L1 adipocytes [124].

### 3.4. Insulin Secretion and Protection of Pancreatic β-Cells

**Figure 3:**
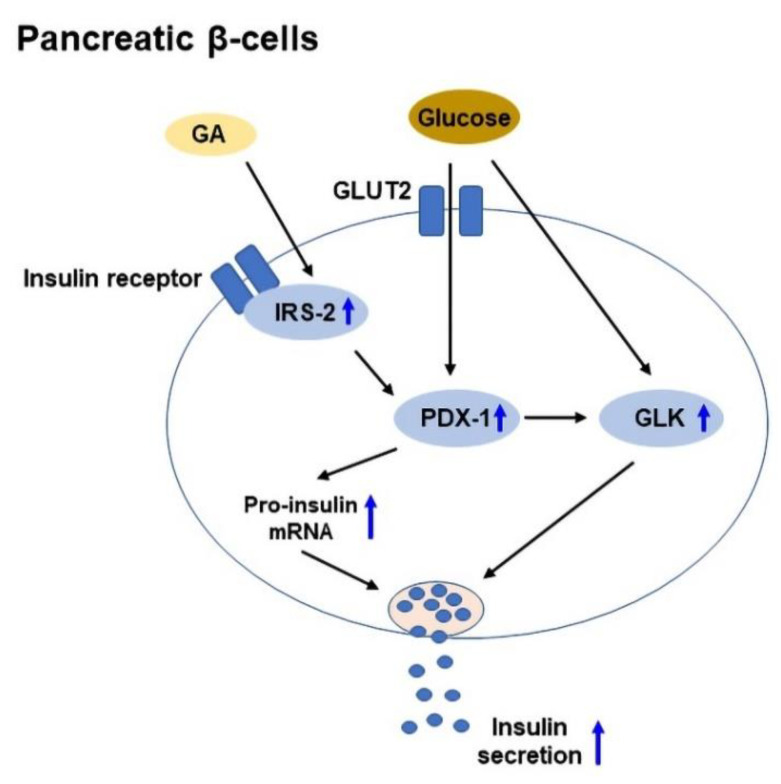
The mechanisms of glycyrrhetinic acid (GA)-mediated glucose-stimulated insulin secretion in pancreatic β-cells. GA upregulates IRS-2, PDX-1, and GLK expressions to protect β-cells and increase insulin secretion. GLUT2: Glucose transporter 2; GLK: Glucokinase; IRS-2: Insulin receptor substrate 2; PDX-1: Pancreas duodenum homeobox-1.

Insulin is a hormone, which is produced by pancreatic β-cells in the islets of Langerhans, and it promotes glucose uptake from the blood into tissues. Insulin secretion is regulated by pancreatic β-cells in response to the increase in blood glucose levels [125]. In T2DM, chronic insulin resistance and a progressive decline in β-cell function result in β-cell dysfunction and defect in insulin secretion [126]. IRS-2 plays a vital role in the preservation of β-cell mass [127], while pancreas duodenum homeobox-1 (PDX-1) plays a role in β-cell survival and function [128]. In addition, glucokinase (GLK) acts as a glucose sensor to regulate insulin secretion [129]. GA was shown to enhance glucose-stimulated insulin secretion in isolated mouse islets, and upregulate IRS-2, PDX-1, and GLK expressions to improve β-cell viability [55]. A schematic diagram of GA-mediated glucose-stimulated insulin secretion in pancreatic β-cells is shown in Figure 3.

## 4. GL and Its Derivatives for the Treatment of T2DM Complications

In patients with T2DM, many years of poorly controlled hyperglycemia can lead to multiple complications that mainly affect small blood vessels (microvascular disease) and large blood vessels (macrovascular disease). The microvascular complications include diabetic retinopathy, nephropathy, and neuropathy, while the major macrovascular complications include diabetic cardiovascular and cerebrovascular diseases [130]. Studies showed that GL and its derivatives exerted different protective effects on diabetic microvascular and macrovascular diseases, which will be discussed in more detail in this part.

### 4.1. Diabetic Retinopathy

Diabetic retinopathy is a microvascular disorder, which is caused by high blood glucose levels damaging the back of the eyes (retina), thus leading to blindness [131]. It is mainly associated with inflammatory processes [132], and studies found that GL improved diabetic retinopathy by inhibiting inflammatory responses [31].

High mobility group box 1 (HMGB1) is a nuclear protein that regulates many gene expressions. When it is activated under pathogenic conditions or stress, it is translocated to the cytoplasm and released into the extracellular environment. Then, the extracellular HMGB1 interacts with its receptors RAGE and toll-like receptor 4 (TLR-4) to trigger pro-inflammatory responses, including interleukin (IL)-1β, IL-6, and TNF-α secretion [133]. It plays a vital role in diabetic retinopathy, and is one potential upstream regulator of TNF-α affecting insulin resistance [134,135] and exhibiting angiogenic effects [136]. Its level is higher in the vitreous fluid of proliferative diabetic retinopathy patients when compared with non-diabetic patients [43,137]. GL, a HMGB1 inhibitor from natural source, suppresses HMGB1 activities through direct binding to HMGB1 [138]. It was found that GL inhibited HMGB1 expression and reduced TNF-α secretion to protect the retina against ischemia/reperfusion-induced damage. In addition, GL was shown to increase IR and IRS-1 phosphorylation in high glucose-induced primary human retinal endothelial cells, thus improving IR signaling [31]. Moreover, high glucose induced the upregulation of HMGB1 and signal transducer and activator of transcription 3 (STAT-3) phosphorylation in human retinal Müller glial cells. In contrast, GL could counteract this effect [139].

### 4.2. Diabetic Nephropathy

Diabetic nephropathy, a major cause of end-stage renal disease, is caused by high blood glucose levels damaging the blood vessel clusters in the kidneys that filter waste in the blood. The development and progression of diabetic nephropathy are associated with some signaling pathways, including oxidative stress, inflammatory responses, activation of transforming growth factor-β (TGF-β) 1, connective tissue growth factor, and MAPK pathway [140]. GL and its derivatives exerted protective effects on diabetic nephropathy by inhibiting oxidative stress [39,141,142], mitochondrial dysfunction [141] and inflammatory responses [40,141], and preventing renal fibrosis [39,143].

Evidence shows that AMP-activated protein kinase (AMPK) downregulation by high glucose is associated with various disturbances during diabetic microvascular complications [144], while sirtuin 1 (SIRT1), a NAD^+^-dependent class III protein deacetylase, is activated to exert cytoprotective effects by inhibiting inflammation, oxidative stress, and apoptosis [145,146]. The activation of SIRT1 results in the downregulation of HMGB1 [43]. A study showed that GL protected NRK-52E renal tubular epithelial cells against high glucose-induced oxidative stress and cell proliferation through the upregulation of AMPK and SIRT1 expressions, and downregulation of TGF-β1 expression [142]. GL was also shown to decrease blood albumin/creatinine levels, reduce reactive oxygen species production, and attenuate renal fibrosis and apoptosis in the kidney via activating AMPK/SIRT1/peroxisome proliferator-activated receptor-gamma co-activator 1α (PGC-1α) signaling in *db/db* mice [39]. In addition, TGF-β1 plays a vital role in tissue fibrosis by enhancing matrix protein synthesis, suppressing matrix degradation, and altering cell-cell interaction [147], and alpha-smooth muscle actin (α-SMA), a marker protein in smooth muscle cells and myofibroblasts that is often used to identify pathological fibroblasts [148]. A study found that GL protected against renal fibrosis in *db/db* mice by reducing the expressions of TGF-β1 and α-SMA in the kidney [39]. Moreover, GL was found to inhibit HMGB1 expression to reduce inflammatory responses and alleviate the progression of diabetic nephropathy, which was through the downregulation of TLR-4, nucleotide-binding domain leucine-rich repeat and pyrin domain-containing receptor 3 (NLRP3), IL-1β expressions and inhibition of nuclear factor kappa B (NF-κB) pathway in the kidney of Zucker diabetic fatty rats [40]. Another study found that GA could suppress the secretion of connective tissue growth factor and collagen IV in high glucose-exposed mesangial cells, indicating that GA could be used for the prevention of mesangial fibrosis that could lead to diabetic nephropathy [143]. Furthermore, 18α-GA reduced mitochondrial dysfunction and oxidative stress through enhancing adenosine triphosphate (ATP) production, suppressing MAPK pathway and activating nuclear factor-erythroid 2-related factor 2 (Nrf-2) pathway in fructose-exposed HK2 proximal tubular epithelial cells, respectively. Moreover, it alleviated inflammation through the inhibition of NF-κB pathway [141]. Meanwhile, it protected against fructose-induced renal injury in mice through alleviating oxidative stress [141].

### 4.3. Diabetic Neuropathy

Diabetic neuropathy is one type of nerve damage that is caused by diabetes. The development and progression of diabetic neuropathy are associated with several factors, including persistent hyperglycemia, microvascular insufficiency, oxidative and nitrosative stress, defective neurotropism, and autoimmune-mediated nerve destruction [149,150]. GL was shown to exert protective effects on diabetic neuropathy by alleviating hyperalgesia and inflammation [41].

GL targeted HMGB1 to ameliorate thermal hyperalgesia in Zucker diabetic fatty rats, in which it alleviated neuroinflammation with decreasing expressions of NLRP3, TLR4, and HMGB1 in the dorsal root ganglia [41]. In addition, it inhibited the expression of HMGB1 in the spinal cord dorsal horn, which is released from the neuronal cells to the cytoplasm or the extracellular space. C-X-C chemokine receptor type 4 (CXCR4) is widely expressed in the sensory neurons of the peripheral and central nervous system, and contributes to the pain signaling in both systems. Its increased expression in the dorsal root ganglia neurons of diabetic rats was reduced after GL treatment [41]. Moreover, AG was shown to prevent high glucose-induced cytotoxic effect, apoptosis, and mitochondrial fragmentation through downregulating HMGB1 expression and NF-κB pathway in SH-SY5Y neuroblastoma cells [151]. Furthermore, aluminum plus fructose could induce hyperinsulinemia and insulin resistance. GL ameliorated aluminum-induced neurotoxicity and memory deficit through anti-inflammatory and anti-oxidative effects on the brain tissue of insulin-resistant rats [29]. In addition, it significantly alleviated insulin resistance in aluminum-induced nephrotoxic insulin-resistant rats, and reduced renal oxidative stress and inflammatory responses and kidney dysfunction [152]. Moreover, GL protected against aluminum-induced renal injury via the inhibition of TLR-4/NF-κB signaling pathway [152].

### 4.4. Diabetic Cardiovascular Disease

High blood glucose levels from diabetes can damage blood vessels and nerves and affect the heart, which can eventually lead to diabetic cardiovascular disease over time [153]. The risk factors of diabetic cardiovascular disease include obesity, hypertension, and dyslipidemia, which are common in patients with T2DM. Moreover, several factors, including increased oxidative stress, inflammation, endothelial dysfunction, and insulin resistance, are often present in patients with T2DM and may directly contribute to the development of diabetic cardiovascular disease [154,155]. GL was shown to exert protective effects on diabetic cardiovascular disease through inhibiting oxidative stress [36] and inflammation [36,156,157,158], alleviating cardiomyocyte atrophy [36], ameliorating endothelial dysfunction [156], and protecting against apoptosis in heart tissue [156].

HMGB1 level was shown to be increased in diabetic patients and *db/db* mice, and it impaired endothelium-dependent relaxation, an initial critical step that leads to various vascular complications in patients with diabetes, through TLR-4/endothelial nitric oxide synthase (eNOS) pathway. GL could attenuate endothelium-dependent relaxation impairment through eNOS-dependent pathway in the aortas of mice [157]. In addition, GL treatment reduced serum HMGB1 level and upregulated eNOS expression in recombinant HMGB1-treated aortas of *db/db* mice. Zucker diabetic fatty rats with myocardial remodeling were treated with GL, and it ameliorated cardiomyocyte atrophy, which was evidenced by counteracting the alterations of connexin43 (Cx43), troponin-I (TnI), and voltage-gated sodium channel (Na_v_1.5) protein expressions in the diabetic heart tissue. Moreover, GL alleviated inflammatory responses via the inhibition of CXCR4/stromal cell-derived factor 1 (SDF1) and TGF-β/p38 MAPK signaling pathways, and improved oxidative stress via the activation of Nrf-2 signaling pathway and downregulation of RAGE expression in the diabetic heart tissue [36]. In addition, GL exerted cardioprotective effects in Zucker diabetic fatty rats with myocardial remodeling by decreasing collagen deposition and apoptosis in the heart tissue, and inhibiting NF-κB signaling pathway to suppress inflammatory responses in the cardiac tissue [36,158]. AGE was found to trigger and aggravate endothelial damage in diabetic vascular complications [159]. A study found that GL protected against AGE-induced endothelial dysfunction in human umbilical vein endothelial cells (HUVECs) through anti-apoptotic, anti-inflammatory, and anti-oxidant activities, via inhibiting RAGE/NF-κB pathway, suggesting that GL can be used for the prevention and treatment of diabetic vascular complications [156]. Furthermore, 18α-GA, as a gap-junction inhibitor, attenuated acetylcholine-induced endothelium-derived hyperpolarizing factor-type relaxation in the mesenteric arteries of T2DM rats [160].

### 4.5. Diabetic Cerebrovascular Disease

Diabetic cerebrovascular diseases are cerebral vascular damage, which is induced by diabetes with high levels of glucose and fat, thus leading to intracranial large and small vessel diseases [161]. Hyperglycemia can promote damage following cerebrovascular disorders, which are associated with oxidative stress, impaired leukocyte function, increased blood-brain barrier permeability, and inflammatory responses [162]. GL was shown to exert protective effects on diabetic cerebrovascular disease by inhibiting inflammation and ameliorating hippocampal microglial and astrocyte activation [37,38].

GL inhibited HMGB1 to protect against hippocampal inflammation and cognitive deficits in high-fat-high-fructose diet-induced obese rats, in which GL prevented HMGB1 translocation from the nucleus to the cytoplasm, suppressed TLR-4 and NF-κB expressions in the hippocampus, reduced plasma and hippocampal IL-1β, TNF-α and IL-6 levels, and attenuated hippocampal microglia and astrocyte activation [37]. Moreover, GL alleviated cerebral ischemia/reperfusion injury in HFD plus STZ-induced diabetic mice through reducing infarct volume, brain edema and neuronal loss, suppressing microglial activation, and inhibiting pro-inflammatory responses, via the downregulation of HMGB1, TLR-4, NF-κB, IL-1β, IL-6, and TNF-α expressions [38].

## 5. Clinical Use of GL and Its Derivatives in T2DM and Its Comorbidities

GL and its derivatives have been shown to exert remarkable therapeutic effects on T2DM and its complications. However, the practical use of GL and its derivatives in T2DM and its complications is limited due to insufficient clinical trials. To date, only GL and DG have been tested in clinical trials for the treatment of T2DM and its comorbidities (Table 1). A 24-week clinical study was conducted to investigate the effects of the combination of metformin and DG enteric-coated capsule (DGEC) for the treatment of non-alcoholic fatty liver disease (NAFLD) in patients with T2DM. One hundred and sixty-three patients were randomized into three groups, metformin treatment (50 patients), DGEC treatment (50 patients), and metformin plus DGEC treatment (46 patients). This study found that metformin plus DGEC treatment had better effects than metformin alone or DGEC alone in lowering metabolic parameters, the levels of liver enzymes and lipids, and alleviating hepatic fibrosis [163]. Similarly, another 6-month clinical study was also conducted to investigate the combined effects of DGEC and metformin for the treatment of NAFLD in T2DM patients. Seventy-six patients were randomized into two groups, acarbose plus simvastatin (38 patients), and metformin plus DGEC (38 patients). They found that the combination of DGEC and metformin improved metabolic parameters, hepatic function, and lipid profile in patients with NAFLD and T2DM [164].

Reactive perforating collagenosis is a rare skin disorder of transepidermal elimination. A study reported on a 73-year-old T2DM female patient with acquired reactive perforating collagenosis. This patient had itchy papules on her back and lower limbs for 3 months and has been diagnosed as a T2DM patient for 15 years. Her blood glucose level was controlled, and improvement was achieved after treatment with topical application of corticosteroids and oral anti-histamine drug along with GL tablets, suggesting that this might be a good therapy to treat acquired reactive perforating collagenosis [165]. In addition, testosterone deficiency is prevalent in T2DM male patients with metabolic syndrome [167]. A clinical study was conducted to investigate the effects of GL on serum testosterone concentrations in T2DM male patients with chronic hepatitis. Thirty-nine male patients were randomized into two groups, without GL treatment (21 patients), and with GL treatment (18 patients). GL was treated for more than 1 year, which decreased serum testosterone concentrations, and they pointed out that the reduction in serum testosterone concentrations might cause insulin resistance [166]. To date, there are limited clinical trials studying the role of GL or derivatives in T2DM and its comorbidities, and these results reveal that GL and its derivatives might have potential therapeutic effects for T2DM due to its anti-hyperglycemic property and improvement in metabolic syndromes. However, there is still a lack of solid clinical evidence to support the safety and effectiveness of GL and its derivatives in T2DM treatment; therefore, this highlights the urgent need for more clinical trials for the treatment of T2DM and its complications.

## 6. Conclusions and Future Perspectives

Over the past decades, the incidence rate of T2DM is increasing rapidly, and T2DM is causing a serious burden to individuals and society worldwide. Traditional Chinese medicine is increasingly attracting the attention, interests, and acceptance of pharmaceutical companies due to low toxicity and high efficacy. GL and its derivatives are major bioactive compounds derived from licorice. They have potent anti-diabetic effects on T2DM and its complications, including lowering blood glucose and insulin levels, improving insulin resistance and glucose tolerance, regulating lipid metabolism, and enhancing insulin secretion. Due to the poor bioavailability of GL, it has been formulated as nanoparticles or conjugated with various metals. Moreover, GL-loaded nanoparticles can reduce blood glucose levels and improve lipid profile, and the dosages used in nanoparticles are only one-quarter of the pure GL form; therefore, the nanoparticles are more potent than the pure compound of GL. Furthermore, GL and DGEC have been studied in clinical trials for the treatment of diabetic comorbidities, indicating their potential use as therapeutic drugs. However, there are limited clinical trials for the treatment of T2DM. Therefore, future direction should be focused on the safety evaluation studies and phase II clinical trials for the development of GL and its derivatives as potential drugs of T2DM and its complications.

## Figures and Tables

**Table 1:** A summary showing the clinical trial data of glycyrrhizic acid (GL) and its derivatives for the management of type 2 diabetes mellitus (T2DM) and its comorbidities.

Drug Name	Trial Design	Trial Length	Sample Size	Patients Recruited	Dosage	Clinical Outcome	Ref.
Diammonium glycyrrhizinate enteric-coated capsule (DGEC)	Randomized controlled trial	24 weeks	146	Patients with T2DM and non-alcoholic fatty liver disease (NAFLD)	Group 1, Metformin alone (500 mg, 3 times daily); Group 2, DGEC alone (450 mg, 3 times daily); Group 3, Metformin (500 mg, 3 times daily) plus DGEC (450 mg, 3 times daily)	Enhanced hypoglycemic action of metformin, including lowering metabolic parameters, the levels of liver enzymes, and lipid levels. Ameliorated hepatic fibrosis.	[163]
DGEC	Randomized uncontrolled trial	6 months	76	Patients with T2DM and NAFLD	Group 1, Acarbose (50–100 mg, 3 times daily) plus Simvastatin (10 mg, once daily); Group 2, Metformin (0.5–1 g, 2 times daily) plus DGEC (50–150 mg, 3 times daily)	Improved metabolic parameters, hepatic function, and lipid profile.	[164]
GL tablets	Single-patient trial	2 months	1	Patients with T2DM and acquired reactive perforating collagenosis	Topical application of corticosteroids (2 times daily), oral anti-histamine drug (once daily), GL tablets (3 times daily)	Blood glucose level was controlled, and skin was improved.	[165]
GL	Randomized controlled trial	>1 year	39	Patients with T2DM and chronic hepatitis	GL (240–525 mg, once weekly)	Decreased serum testosterone concentrations.	[166]

## Data Availability

Not applicable.
